# Structural constraints on pronoun binding and coreference: evidence from eye movements during reading

**DOI:** 10.3389/fpsyg.2015.00840

**Published:** 2015-06-23

**Authors:** Ian Cunnings, Clare Patterson, Claudia Felser

**Affiliations:** ^1^School of Psychology and Clinical Language Sciences, University of ReadingReading, UK; ^2^Potsdam Research Institute for Multilingualism, University of PotsdamPotsdam, Germany

**Keywords:** pronoun resolution, memory retrieval, quantification, eye movements, reading, English

## Abstract

A number of recent studies have investigated how syntactic and non-syntactic constraints combine to cue memory retrieval during anaphora resolution. In this paper we investigate how syntactic constraints and gender congruence interact to guide memory retrieval during the resolution of subject pronouns. Subject pronouns are always technically ambiguous, and the application of syntactic constraints on their interpretation depends on properties of the antecedent that is to be retrieved. While pronouns can freely corefer with non-quantified referential antecedents, linking a pronoun to a quantified antecedent is only possible in certain syntactic configurations via variable binding. We report the results from a judgment task and three online reading comprehension experiments investigating pronoun resolution with quantified and non-quantified antecedents. Results from both the judgment task and participants' eye movements during reading indicate that comprehenders freely allow pronouns to corefer with non-quantified antecedents, but that retrieval of quantified antecedents is restricted to specific syntactic environments. We interpret our findings as indicating that syntactic constraints constitute highly weighted cues to memory retrieval during anaphora resolution.

## Introduction

The successful interpretation of anaphoric elements during language comprehension involves forming dependencies between constituents that may span several words or sentences. Anaphora resolution thus provides a key test case for studying the memory system that subserves language comprehension, as the correct interpretation of anaphoric constituents crucially relies on the retrieval of a particular item, the antecedent, from memory. A growing number of studies have investigated how syntactic and non-syntactic factors combine to cue the retrieval of an antecedent during the resolution of different types of anaphora (Badecker and Straub, 2002; Sturt, 2003; Xiang et al., 2009; Clackson et al., 2011; Cunnings and Felser, 2013; Dillon et al., 2013; Chow et al., 2014; Clackson and Heyer, 2014; Cunnings and Sturt, 2014; Patterson et al., 2014). Most previous research has investigated constraints on the resolution of reflexives and object pronouns, where syntactic constraints (e.g., binding conditions A and B; Chomsky, 1981) restrict memory retrieval to an antecedent in a particular syntactic domain. Research on real-time pronoun resolution has investigated the extent to which such syntactic constraints interact with other sources of information, such as discourse prominence and gender/number congruence, to guide the retrieval of a particular antecedent. While some have claimed that syntactic constraints act as “hard constraints” that restrict memory retrieval to syntactically licit antecedents (e.g., Dillon et al., 2013; Chow et al., 2014), others argue that syntactic constraints are violable and interact with other sources of information to cue antecedent retrieval (e.g., Badecker and Straub, 2002).

While binding conditions A and B have been well studied, to date little research has investigated the time-course of the application of syntactic constraints on the interpretation of pronouns linked to quantified and non-quantified antecedents as in (1) and (2) respectively.

**Table:** 

(1a)	The man heard that every boy at school said that he was happy.
(1b)	The man who every boy at school heard said that he was happy.
(2a)	The man heard that the boy at school said that he was happy.
(2b)	The man who the boy at school heard said that he was happy.

In (1a), the subject pronoun *he* can refer to either the matrix subject *the man* or the quantified antecedent *every boy*. In (1b) however, when *every boy* appears inside a relative clause, it is not possible for the pronoun to be bound by it. Note that this is not an absolute restriction on antecedents inside relative clauses however, as the pronoun can freely refer to the non-quantified antecedent *the boy* in both (2a) and (2b). This contrast between quantified and non-quantified antecedents thus provides a particular challenge for memory retrieval mechanisms during language processing. For example, if retrieval operations disfavored antecedents inside relative clauses this would ensure that syntactically illicit quantified antecedents, as in (1b), are not retrieved, but would also rule out perfectly licit non-quantified antecedents as in (2b). Conversely, if subject pronouns routinely trigger retrieval of antecedents inside relative clauses, syntactically illicit quantified antecedents may be retrieved. Instead, successful pronoun interpretation requires selective retrieval of antecedents inside relative clauses, but this is dependent on the properties of the to-be-retrieved material.

The aim of the current study was to investigate the retrieval of quantified and non-quantified antecedents during pronoun resolution to further examine how syntactic constraints on memory retrieval are implemented during real-time anaphora resolution. To this end, we conducted an offline judgment task and three online reading experiments investigating pronoun resolution with quantified and non-quantified antecedents. We begin below by discussing theoretical accounts of the contrast between quantified and non-quantified antecedents as exemplified in (1) and (2), before discussing implications of this contrast for models of memory retrieval during language processing in more detail.

## Background

### Variable binding and coreference assignment

In linguistic theory, it has been claimed that pronoun resolution can be achieved in different ways. Although theoretical accounts differ in their precise nature, a core idea is that pronouns can be resolved either in the discourse representation, via coreference assignment, or in logical syntax, via variable binding (e.g., Evans, 1980; Bosch, 1983; Reinhart, 1983; Reuland, 2001, 2011). Coreference assignment involves linking a pronoun to a referential antecedent in the discourse, as in the case of linking the pronoun *he* to either of the antecedents (*the man* or *the boy*) in (2). Quantified phrases (QPs), as in (1), however do not refer to a single individual in the discourse, and a pronoun linked to a QP co-varies in interpretation with the quantifier. Pronouns linked to QPs are thus said to involve variable binding rather than coreference assignment.

A long-standing observation in the linguistics literature is that variable binding is only possible in certain syntactic configurations. This restriction has traditionally been characterized in terms of c-command. C-command refers to a relationship between constituents in the phrase structure representation of a sentence based on the notion of hierarchical dominance. In the standard definition, a constituent c-commands its sister constituents and any constituents that these dominate (Reinhart, 1983). Variable binding is only possible between a pronoun and an antecedent that c-commands it (see e.g., Reuland, 2001, 2011). As such, in (3a), when the QP *every boy* c-commands the pronoun, the pronoun can be bound by it, while in (3b), when the QP does not c-command the pronoun, variable binding between the pronoun and QP is not possible. Coreference assignment to non-quantified determiner phrases (DPs) is not contingent on c-command however, and as such the pronoun can corefer with the referential antecedent *the boy* in both (2a) and (2b).

### Memory retrieval during language processing

Recent psycholinguistic research has motivated a cue-based model of memory retrieval during language processing (McElree, 2000; McElree et al., 2003; Lewis et al., 2006). In cue-based content-addressable models, retrieval is achieved by matching a set of retrieval cues with the contents of all items in memory in parallel. The item in memory that provides the best match to these cues becomes most highly activated and will thus be retrieved. The distinction between variable binding and coreference assignment has a number of implications for models of memory retrieval during language processing.

One theoretical implication relates to how the c-command constraint on variable binding is implemented in content-addressable memory. Content-addressable models are well-suited to utilize feature-based cues that target intrinsic properties of to-be-retrieved material. For example, it is straightforward to implement a [+masculine] feature for masculine pronouns to cue retrieval of a masculine antecedent. However, the c-command constraint on variable binding may be more difficult to implement, as it involves access to information about the *relation* between two items in memory (the pronoun and antecedent), rather than accessing an intrinsic *feature* of the antecedent (for discussion, see Kush, 2013; Kush et al., 2015). The primary aim of the current study was to investigate *if* the c-command constraint on variable binding restricts antecedent retrieval, rather than the question of *how* it is implemented. We do however return to this issue in the General Discussion.

A second implication that the distinction between variable binding and coreference assignment has for models of memory retrieval during language processing relates to how pronouns that are ambiguous with regards to variable binding and coreference assignment are resolved. Research in theoretical linguistics has claimed that syntactic variable binding is preferred over coreference assignment. Reuland (2001, 2011), for example, proposed an economy principle which predicts that variable binding should be computed before coreference assignment is attempted (see also Koornneef, 2008). This predicts that variable binding antecedents should preferentially be retrieved before coreference antecedents. Cunnings et al. (2014) tested this prediction in two reading experiments. They manipulated gender congruence between a pronoun and two potential antecedents in the discourse, and monitored participants' eye-movements as they read sentences as in (3).

**Table:** 

(3a)	Every soldier who knew that James/Helen was watching was convinced that he/she should wave as the parade passed.
(3b)	It looked to James/Helen that every soldier was completely convinced that he/she should wave as the parade passed.

In their Experiment 1, exemplified in (3a), gender congruence was manipulated between the pronoun and a c-commanding QP (*every soldier*), and between the pronoun and a non c-commanding but linearly closer proper name coreference antecedent (*James/Helen*). They hypothesized that if variable binding is computed before coreference assignment, when participants encounter the pronoun, the c-commanding QP antecedent should be preferentially retrieved. In this case, the gender of the c-commanding QP should affect reading times at a point in time before the gender of the proper name. However, in contrast to this prediction, they observed that reading times at and shortly after the pronoun were longer when the pronoun mismatched in gender with the proper name antecedent, and were not significantly affected by the gender of the QP. This suggests that the proper name antecedent, rather than the QP, was preferentially retrieved upon encountering the pronoun. In their Experiment 2 however, exemplified in (3b), when the QP was linearly closer to the pronoun than the proper name antecedent, reading times at and shortly after the pronoun were reliably longer when the QP mismatched in gender with the pronoun. Together, these results indicate there is no overall preference for either variable binding or coreference assignment. For variable binding antecedents to be retrieved additional factors, such as antecedent recency, need to favor the QP antecedent.

A third issue relates to how the c-command constraint on variable binding, however it is implemented, interacts with other cues to antecedent retrieval. Cunnings et al. only investigated cases in which variable binding antecedents were syntactically licit, and did not test sentences containing QPs that did not c-command the critical pronoun. A key prediction of cue-based models is similarity-based interference (see e.g., Lewis et al., 2006; Van Dyke and Johns, 2012). As retrieval involves the matching of a set of retrieval cues with all items in memory in parallel, a distractor item that partially matches the retrieval cues may sometimes be retrieved instead of the intended retrieval target. This leads to the possibility that a QP antecedent that does not c-command a pronoun may occasionally be retrieved even though this dependency is ungrammatical.

Attraction effects in subject-verb agreement are a key example of such interference effects during language processing. For example, Wagers et al. (2009) reported longer reading times for sentences containing ungrammatical compared to grammatical subject-verb agreement (e.g., *the key to the cabinet was rusty* vs. *the key to the cabinet were rusty*). This ungrammaticality effect was however reliably attenuated when the structurally illicit distractor matched the agreement marking of the critical verb (e.g., *the key to the cabinets were rusty*). This attraction effect provides good evidence that structural cues (e.g., [+phrasal head]) and agreement (e.g., [+plural]) are equally weighted cues that combine to guide retrieval during subject-verb agreement. When no item in memory fully matches the cues at retrieval, a partially matching distractor can sometimes be retrieved. We will refer to this pattern of results as *facilitatory* interference, as the processing of ungrammatical sentences is facilitated by a partially matching distractor.

Although facilitatory attraction effects are well attested for subject-verb agreement, a number of studies have failed to observe this specific pattern of interference during anaphora resolution (e.g., Sturt, 2003; Xiang et al., 2009; Dillon et al., 2013; Chow et al., 2014; Cunnings and Sturt, 2014). Sturt, for example, manipulated gender congruence between a reflexive and two antecedents in a piece of discourse (e.g., *Jonathan/Jennifer remembered that the surgeon had pricked himself/herself with a used syringe needle*) and observed that while first-pass reading times at the reflexive were reliably longer when the structurally licit antecedent *the surgeon* mismatched in stereotypical gender with the reflexive, the gender of the structurally illicit antecedent (*Jonathan/Jennifer*) did not affect reading times in this measure. Results such as these have led some to claim that while equally weighted syntactic and agreement cues combine to guide retrieval for subject-verb agreement, anaphora resolution is guided by syntactic “hard constraints” that restrict retrieval to syntactically licit antecedents (Dillon et al., 2013; Chow et al., 2014). Although the question of whether or not structurally illicit antecedents are always ignored during anaphora resolution is debated (e.g., Badecker and Straub, 2002; Cunnings and Felser, 2013; Clackson and Heyer, 2014), the contrast in attraction effects observed for agreement and anaphora suggests these dependencies implement agreement cues in different ways. For anaphora, syntactic constraints appear to be more strongly weighted cues to retrieval than gender/number congruence.

Syntactic constraints on reflexives could potentially be implemented as highly weighted cues that trigger retrieval of an antecedent within a particular syntactic domain (e.g., the same clause as the reflexive; see Dillon et al., 2013). However, constraints on quantified and non-quantified antecedents are difficult to implement in this way, as it is not the case that antecedents within a particular syntactic domain (e.g., a relative clause) are categorically ruled out. Rather, sensitivity to constraints on variable binding and coreference assignment require retrieval operations to be able to selectively retrieve antecedents that do not c-command pronouns depending on their quantificational status. The contrast between variable binding and coreference assignment thus provides a unique challenge to memory retrieval operations during language processing, which may leave variable binding more susceptible to facilitatory interference than has been observed for other types of anaphora, such as reflexives.

We are aware of only one study that has investigated the potential for facilitatory interference during the resolution of bound variable anaphora. Kush et al. (2015) recorded participants' eye-movements as they read sentences as in (4).

**Table:** 

(4a)	The troop leaders that the boy/girl scout had no respect for had scolded her after the incident at scout camp.
(4b)	The troop leaders that no boy/girl scout had respect for had scolded her after the incident at scout camp.
(4c)	The troop leaders were sure no boy/girl scout was afraid that she would be scolded after the incident at scout camp.

In (4a), the only syntactically licit antecedent for the pronoun *her* is the coreference antecedent *the boy/girl*. In (4b), the pronoun has no syntactically licit antecedent as the quantified phrase (*no boy/girl*) does not c-command it. Kush et al. hypothesized that if the pronoun triggers retrieval of the coreference antecedent in (4a), a gender mismatch effect should be observed, with longer reading times for gender mismatching (*the boy*) than gender matching (*the girl*) antecedents. If antecedent retrieval respects the c-command constraint, this contrast between gender matching (*no girl scout*) and gender mismatching (*no boy scout*) antecedents should not be observed in (4b). If the c-command constraint does not restrict antecedent retrieval however, Kush et al. hypothesized that the gender mismatch effect should be observed in both (4a) and (4b), as evidence of facilitatory interference. During first-pass processing at the pronoun Kush et al. observed a gender mismatch effect in (4a) but not (4b), suggesting the c-command constraint on variable binding restricts the early stages of antecedent retrieval. They did observe gender mismatch effects in (4c) however, when the quantified phrase c-commanded the pronoun. Kush et al interpreted these results as indicating that pronouns trigger retrieval of both c-commanding quantified phrases and non c-commanding coreference antecedents, but not non c-commanding quantified antecedents, suggesting that the c-command constraint restricts antecedent retrieval.

Against this background, the aim of the current study was to further investigate the implementation of the c-command constraint on variable binding during anaphora resolution. While Kush et al. compared antecedent retrieval for c-commanding and non c-commanding quantified antecedents in different sentence structures with different (subject and object) pronouns, we investigated variable binding and coreference resolution in maximally similar sentences with identical (subject) pronouns across four experiments. We also tested the universal quantifier *every* rather than the negative quantifier *no*. Together with the study reported by Kush et al., the current experiments provide a systematic examination of how constraints on retrieving quantified phrases and referential antecedents during anaphora resolution are implemented during language processing. Experiment 1 was an offline task that tested the extent to which naïve participants are sensitive to the c-command constraint on variable binding in an untimed task. Experiments 2–4 were online reading studies in which participants' eye-movements were monitored. Experiments 2–3 contrasted the retrieval of quantified and non-quantified referential antecedents in order to test the extent to which variable binding and coreference antecedents are retrieved in c-commanding and non c-commanding configurations. Experiment 4 tested the extent to which the c-command restriction on variable binding acts as a “hard constraint” on antecedent retrieval.

## Experiment 1

Experiment 1 used a sentence judgment paradigm to assess sensitivity to the c-command constraint on variable binding in an untimed offline task. The materials consisted of sentences as in (5), which manipulate the factor “c-command” to test whether participants are willing to link pronouns to QPs in different syntactic configurations.

**Table:** 

(5a)	*C-commanding QP*
	The surgeon suggested that every man on the waiting list definitely realized that he needed some help.
(5b)	*Non c-commanding QP*
	The surgeon who every man on the waiting list suggested definitely realized that he needed some help.

In (5a), the QP *every man* c-commands the pronoun *he* and as such the pronoun can be bound by the QP via variable binding. In (5b) however, the QP appears inside a relative clause and as such does not c-command the pronoun. In this case, the pronoun can only refer to the matrix subject *the surgeon*. We expect native English speakers to be sensitive to the c-command constraint on variable binding in this offline task. That is, participants should consider the QP as a possible antecedent for the pronoun in (5a) but not (5b).

### Methods

#### Participants

32 native English speakers (17 males, mean age 21; range 18–30) from the University of Edinburgh community either received course credit or a small payment for taking part in Experiment 1^1^. All participants in Experiment 1, and Experiments 2–4, provided written, informed consent before the experiment began. Ethical approval for all experiments was granted by the Department of Psychology Research Ethics Committee at the University of Edinburgh.

#### Materials

Materials consisted of 16 experimental items constructed as in (5). In each item, the pronoun matched in definitional gender with the QP antecedent. The pronoun also always matched in stereotypical gender with the matrix subject to ensure that the texts were felicitous. The materials manipulated the factor “c-command” in two conditions, such that the QP either c-commanded or did not c-command the QP. A full list of experimental items is provided in Appendix A. In addition to the experimental items, 24 filler items were also constructed, some of which also contained pronouns but others which did not.

#### Procedure

The experimental and filler items were presented to participants as a questionnaire in Microsoft Word. A question appeared under each text with two possible answers. For the experimental items, the question always probed the interpretation of the pronoun. In (4), for example, the question was “Who does ‘he’ refer to?” with “(A) the surgeon” and “(B) every man” as possible answers. Participants provided a response by selecting one of five options from a drop-down menu that appeared beside each text. Possible responses were “(A) strongly preferred”, “(A) mildly preferred”, “(A) or (B) equally likely”, “(B) mildly preferred” or “(B) strongly preferred”. Across the 16 experimental items, the matrix subject and QP antecedents each appeared as options “(A)” and “(B)” an equal number of times. Fillers that did not include pronouns consisted of complex (ambiguous and unambiguous) sentences containing elliptical gaps. Two paraphrases, (A) and (B), were provided as answers which participants had to choose between using the same scale as in the experimental items.

The experimental and filler items were pseudo-randomized such that no two experimental items appeared next to each other. Items were spread across two presentation lists in a Latin-square design. Forward and reverse orders of each list were presented to the same number of participants. Participants were instructed to simply read each sentence and provide an answer to the questions using the drop-down menu.

### Results

Responses were coded from −2 to 2, with −2 meaning “QP strongly preferred” and 2 meaning “DP strongly preferred.” A score of 0 indicated either antecedent was equally likely, while −1 and 1 indicated a mild preference for the QP and DP respectively. The average rating in the c-commanding QP condition was −0.16 (SD 1.62) and in the non c-commanding condition 1.38 (SD 1.14). A pairwise comparison indicated that scores were significantly higher in the non c-commanding QP condition than the c-commanding QP condition [*t*_1(31)_ = 8.19, *p* < 0.001; *t*_2(15)_ = 11.80, *p* < 0.001]. This indicates that the DP antecedent was chosen more often when the QP did not c-command the pronoun compared to when it did. One sample *t*-tests indicated that the average scores in the c-command condition did not differ significantly from 0 [*t*_1(31)_ = 0.90, *p* = 0.374; *t*_2(15)_ = 1.23, *p* = 0.237], but that the scores in the non c-command condition were significantly higher than 0 [*t*_1(31)_ = 11.12, *p* < 0.001; *t*_2(15)_ = 16.46, *p* < 0.001]. This indicates that when the QP c-commanded the pronoun, participants considered either antecedent equally likely, but that the DP was preferred when the QP did not c-command the pronoun.

### Discussion

The results of Experiment 1 align with intuitions from the theoretical linguistics literature. When the QP c-commanded the pronoun, participants were equally likely to interpret the pronoun as referring to either the QP or the DP antecedent. When the QP did not c-command the pronoun, participants preferred to interpret the pronoun as being coreferential with the DP. Experiment 1 thus suggests that naïve participants are sensitive to the c-command restriction on variable binding^2^. Experiment 2 tested how this constraint is implemented during online sentence processing.

## Experiment 2

The aim of Experiment 2 was to investigate the application of the c-command constraint on variable binding during real-time language processing. Participants read a series of texts as in (6) while their eye-movements were monitored. The gender-mismatch paradigm (Sturt, 2003; Kazanina et al., 2007) was used as a diagnostic of dependency formation.

**Table:** 

(6a)	*C-commanding QP, gender match*
	Being in hospital can be quite difficult at times.
	The surgeon saw that every old man on the emergency ward silently wished that he could go a little bit faster.
(6b)	*C-commanding QP, gender mismatch*
	Being in hospital can be quite difficult at times.
	The surgeon saw that every old woman on the emergency ward silently wished that he could go a little bit faster.
(6c)	*Non c-commanding QP, gender match*
	Being in hospital can be quite difficult at times.
	The surgeon who every old man on the emergency ward saw silently wished that he could go a little bit faster.
(6d)	*Non c-commanding QP, gender mismatch*
	Being in hospital can be quite difficult at times.
	The surgeon who every old woman on the emergency ward saw silently wished that he could go a little bit faster.

In (6a,b) the pronoun *he* is c-commanded by the QP *every old (wo)man*. In (6c,d) the pronoun is not c-commanded by the QP, as it appears inside a relative clause. In (6a,c) the QP *every old man* matches the gender of the pronoun, while in (6b,d) the QP *every old woman* does not. If participants attempt to retrieve the c-commanding QP upon encountering the pronoun, we expect to observe a gender mismatch effect such that reading times at or shortly after the pronoun should be longer in gender mismatch condition (6b) than gender match condition (6a). If the c-command constraint restricts antecedent retrieval during processing (Kush et al., 2015), no gender mismatch effect should be observed when the QP appears inside a relative clause, as in (6c,d). If however participants violate the c-command constraint during processing, we can expect to see gender mismatch effects in both (6a,b) and (6c,d). Sensitivity to the c-command constraint is thus diagnosed statistically by an interaction between the main effects of c-command and gender, while main effects of gender would indicate constraint violation.

### Methods

#### Participants

Thirty two native English speakers (8 males, mean age 19; range 17–23) from the University of Edinburgh community with normal or corrected-to-normal vision, and who did not take part in any of the other experiments reported here, took part in Experiment 2.

#### Materials

Twenty four experimental items as in (6) were constructed. A full list can be found in Appendix B. Each item began with a short context sentence that took up one line onscreen. The critical second sentence appeared across two lines, with the line-break always appearing before the adverb [*silently* in (6)] that appeared before the verb preceding the critical pronoun. The matrix subject of the critical sentence always matched the pronoun in stereotypical gender to ensure that a felicitous interpretation of the pronoun was always possible. The critical gender manipulation between the QP and pronoun always involved definitional gender (e.g., *every old man/woman*).

In addition to the experiment items, 60 filler texts were also constructed that included a variety of different constructions, some of which included different types of anaphors. The fillers took up between two and three lines of text onscreen.

#### Procedures

Experimental and filler items were pseudo-randomized such that no two experimental items appeared adjacent to each other and were spread across four presentation lists in a Latin-square design. A different random order of items was presented to each participant. The experiment began with five practice items to familiarize participants with the procedure. All items were presented in Consolas fixed width font and displayed across up to three lines of text onscreen.

Eye movements were recorded using the EYELINK 2000 system, sampling at a rate of 1000 Hz. While viewing was binocular, eye movements were recorded from the right eye only. Each experimental session began with calibration of the eye-tracker on a nine-point grid, and any drift in calibration was compensated for via recalibration between trials if required. Before each trial, participants fixated on a fixation marker above the first word of the trial to be displayed. Upon fixation on this marker, the trial text appeared. Participants read each text silently at their normal reading rate, pressing a button on a control pad once completed. To ensure participants paid attention to the content of the sentences, comprehension questions requiring a yes/no push button response followed two thirds of all trials. The entire experiment lasted approximately 30–45 min in total.

Reading times are reported for four regions of text. The critical *pronoun region* consisted of the subject pronoun and the preceding complementiser (*that he*). We extended the pronoun region to the left of the critical pronoun rather than the right to avoid effects of first-pass processing at the pronoun being mixed with spillover effects at the post-pronoun region. As the perceptual span in English is approximately eight characters to the right of fixation (Rayner, 1998), fixations on the complementiser are likely to involve foveal processing of the pronoun. The *spillover region* comprised the two words after the pronoun (*could go*) while the *prefinal region* consisted of the next two words (*a little*). The *final region* consisted of the rest of the critical sentence (*bit faster*).

Four reading time measures are reported for each region of text. *First pass reading time* is the summed duration of fixations within a region during its first inspection, until it is exited to the left or right, while *regression path duration* is calculated by summing the duration of each fixation, starting with the first fixation when a region is entered from the left, up until but not including the first fixation in a region to the right. In addition to these two *first-pass* processing measures, we also calculated *second pass times*, which included all fixations within a region *after* it has been exited following the first-pass. *Total viewing times*, which sum all fixations in a region, are reported as a global measure of processing load. All trials in which track loss occurred were discarded, and regions which were initially skipped during reading were treated as missing data in the two first-pass measures. For second pass times, trials in which a region was not refixated after the first-pass contributed a second pass time of zero to the calculation of averages. Prior to the calculation of reading time measures an automatic procedure merged short fixations of 80 ms or below that were within one degree of visual arc of another fixation. All other fixations of 80 ms or below, as well as those above 800 ms, were removed. Outliers that were above or below 3.5 standard deviations from a participant's mean reading time for each measure were also removed before analysis.

Analysis was conducted using linear-mixed effects models with crossed random effects for subjects and items (Baayen, 2008; Baayen et al., 2008). For each reading time measure, the analysis included deviation-coded fixed main effects of “c-command” (c-command vs. non c-command), “gender” (match vs. mismatch) and their interaction. Subject and item random intercepts, as well as subject and item random slopes for each fixed effect, were included using a “maximal” random effects structure (Barr et al., 2013). If this maximal model failed to converge, the random effects structure was simplified by removing the random correlation parameters, which for the analyses reported here always led to convergence. For fixed effects, *p*-values were estimated from the *t* distribution (Baayen, 2008, p. 248). In the case of reliable interactions, planned comparisons compared gender mismatch effects separately for the two c-command and two non c-command conditions.

### Results

Overall accuracy to the comprehension questions was 88% (all subjects above 73%), indicating that participants paid attention to the content of the sentences. Track loss accounted for 0.1% of the data and skipping rates for the pronoun, spillover, prefinal and final regions were 26, 5, 19, and 10% respectively^3^. A summary of the reading time data is provided in Table 1. Table 2 provides a summary of the statistical analysis.

**Table 1:** **Reading times in milliseconds for four eye-movement measures at four regions of texts in Experiment 2 (SDs in parentheses)**.

	**First pass reading time**	**Regression path time**	**Second pass time**	**Total viewing time**
**PRONOUN REGION**				
C-commanding QP, gender match	226 (91)	260 (158)	105 (229)	328 (278)
C-commanding QP, gender mismatch	235 (126)	302 (278)	176 (271)	417 (297)
Non c-commanding QP, gender match	256 (131)	303 (230)	142 (240)	409 (287)
Non c-commanding QP, gender mismatch	259 (155)	330 (284)	137 (213)	390 (275)
**SPILLOVER REGION**				
C-commanding QP, gender match	262 (120)	303 (177)	170 (239)	419 (253)
C-commanding QP, gender mismatch	285 (166)	370 (263)	280 (319)	561 (331)
Non c-commanding QP, gender match	275 (170)	345 (354)	213 (284)	497 (356)
Non c-commanding QP, gender mismatch	274 (140)	339 (223)	205 (258)	478 (291)
**PREFINAL REGION**				
C-commanding QP, gender match	255 (155)	424 (638)	180 (230)	406 (263)
C-commanding QP, gender mismatch	266 (159)	531 (902)	188 (255)	440 (286)
Non c-commanding QP, gender match	275 (146)	488 (798)	177 (241)	439 (269)
Non c-commanding QP, gender mismatch	267 (123)	419 (394)	183 (247)	435 (270)
**FINAL REGION**				
C-commanding QP, gender match	287 (160)	1840 (2070)	139 (252)	437 (278)
C-commanding QP, gender mismatch	297 (174)	2288 (2234)	157 (2334)	493 (365)
Non c-commanding QP, gender match	295 (169)	2221 (2592)	138 (2592)	452 (316)
Non c-commanding QP, gender mismatch	290 (178)	2023 (2143)	146 (2143)	460 (349)

**Table 2:** **Summary of statistical analyses for four eye-movement measures at four regions of texts in Experiment 2**.

	**First pass reading time**		**Regression path time**		**Second pass time**		**Total viewing time**	
	**Estimate**	***t***	**Estimate**	***t***	**Estimate**	***t***	**Estimate**	***t***
**PRONOUN REGION**								
C-command	25 (11)	2.392^*^	35 (23)	1.529	2 (17)	0.118	25 (19)	1.298
Gender	3 (12)	0.280	32 (25)	1.272	34 (22)	1.527	35 (27)	1.301
C-command^*^ gender	1 (24)	0.035	14 (48)	0.288	74 (37)	2.012^*^	90 (38)	2.357^*^
**SPILLOVER REGION**								
C-command	2 (12)	0.135	8 (22)	0.359	16 (21)	0.795	1 (23)	0.035
Gender	11 (13)	0.807	28 (22)	1.230	52 (24)	2.162^*^	58 (27)	2.189^*^
C-command^*^ gender	22 (24)	0.925	75 (47)	1.612	117 (41)	2.852^*^	161 (50)	3.216^*^
**PREFINAL REGION**								
C-command	12 (13)	0.911	14 (53)	0.269	4 (17)	0.263	9 (22)	0.419
Gender	2 (12)	0.152	17 (53)	0.313	7 (24)	0.275	18 (26)	0.685
C-command^*^ gender	24 (22)	1.105	203 (118)	1.719^(^^*^^)^	1 (33)	0.027	41 (37)	1.095
**FINAL REGION**								
C-command	3 (12)	0.245	54 (172)	0.313	7 (24)	0.289	1 (23)	0.053
Gender	1 (13)	0.096	124 (163)	0.760	13 (25)	0.525	25 (29)	0.882
C-command^*^ gender	16 (24)	0.671	546 (312)	1.751^(^^*^^)^	9 (38)	0.222	35 (50)	0.699

Estimate = Model Estimate (SE in brackets). (

* ) = p < 0.10,

* = p < 0.05,

** = p < 0.001.

**Figure 1:**
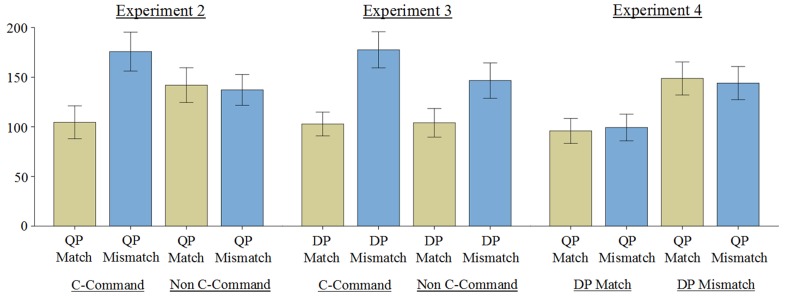
**Second pass times in milliseconds (with standard errors) at the pronoun region in Experiments 2–4**.

At the pronoun region, there was a significant main effect of c-command in first-pass reading times, with reading times being longer in the two non c-command conditions (6c,d) than c-commanding conditions (6a,b). This likely reflects spillover processing as a result of the extra layer of syntactic embedding from the relative clause that appears in conditions (6c,d) but not (6a,b). There were significant c-command by gender interactions in both second-pass and total viewing times. Planned comparisons in both measures indicated that when the QP c-commanded the pronoun, reading times were longer in gender mismatch condition (6b) than gender match condition (6a) (for second-pass times, estimate = 71, SD = 30, *t* = 2.407, *p* = 0.017; for total viewing times, estimate = 80, SD = 31, *t* = 2.586, *p* = 0.010). The same comparisons in the two non c-command conditions were not significant (for both measures, *t* < 1, *p* > 0.651). This pattern of results, with gender mismatch effects in the c-command conditions only, is illustrated for second pass times in Figure 1. These results indicate that readers attempted to link the pronoun to the QP when it c-commanded the pronoun but not when it did not.

At the spillover region, there were significant main effects of gender in both second-pass and total viewing times that were modulated by significant c-command by gender interactions in both measures. Again, planned comparisons in the c-command conditions indicated significantly longer reading times for gender mismatch condition (6b) than gender match condition (6a) (for second-pass times, estimate = 111, SD = 33, *t* = 3.403, *p* < 0.001; for total viewing times, estimate = 134, SD = 35, *t* = 3.920, *p* < 0.001), but there were no significant differences between the two non c-command conditions (for both measures, *t* < 1, *p* > 0.563). These results further indicate that readers retrieved the QP upon encountering the pronoun, but only when the QP c-commanded it.

At the prefinal and final regions there were marginally significant c-command by gender interactions in the regression path times. At the prefinal region, regression path durations were again numerically larger following a gender mismatch in the c-command conditions only, but here neither of the planned comparisons was significant (both *t* < 1.2, both *p* > 0.236). At the final region, regression path durations were marginally longer following gender mismatches in the two c-command conditions (estimate = 405, SD = 225, *t* = 1.801, *p* = 0.073). The same comparison for the two non c-command conditions was not significant (*t* < 1. *p* > 0.470).

### Discussion

The results of Experiment 2 clearly show that readers readily retrieved the QP upon encountering the pronoun, but only when it was a syntactically licit antecedent. At both the critical pronoun and spillover regions, second-pass and total viewing times were longer when the QP mismatched in gender with the pronoun, but only when the QP c-commanded the pronoun. Similar trends were also observed in regression path times at later regions of text. At no point in time did we observe any reliable effect of the gender of the QP on participants' reading times when it did not c-command the pronoun. These results indicate that the c-command constraint on variable binding restricts antecedent retrieval during the resolution of subject pronouns.

One potential counterargument to this interpretation of our results is that the QPs inside relative clauses may have been ignored during retrieval not because of the c-command constraint on variable binding, but rather because antecedents inside relative clauses are comparatively non-discourse prominent. The results of Cunnings et al. (2014) however provide evidence against this interpretation. In their Experiment 1, they observed that readers would readily retrieve a non-quantified coreference antecedent inside a relative clause. This suggests that it is not the case that all antecedents inside relative clauses are ignored during retrieval, but rather they are readily retrieved only when syntactically licit.

However, it remains at least possible that there may have been subtle pragmatic differences between the texts used in Experiment 2 reported here and those used by Cunnings et al. (2014), which may have favored retrieval of the relative clause antecedent in Cunnings et al.'s study but not here. Note also that the coreference antecedent in Cunnings et al. was a proper name, which are known to be particularly discourse prominent (Sanford and Garrod, 1988). The aim of Experiment 3 was to investigate whether the selective retrieval profile observed in the current experiment is truly a result of the c-command constraint on variable binding or results from differences in discourse prominence between c-commanding and non c-commanding antecedents in general.

## Experiment 3

The aim of Experiment 3 was to investigate whether the c-command relationship between antecedent and pronoun affects the possibility of retrieval for non-quantified referential antecedents. The experimental materials used were identical to those from Experiment 2, except that the critical QP was replaced with a non-quantified referential DP as in (7).

**Table:** 

(7a)	*C-commanding DP, gender match*
	Being in hospital can be quite difficult at times.
	The surgeon saw that the old man on the emergency ward silently wished that he could go a little bit faster.
(7b)	*C-commanding DP, gender mismatch*
	Being in hospital can be quite difficult at times.
	The surgeon saw that the old woman on the emergency ward silently wished that he could go a little bit faster.
(7c)	*Non c-commanding DP, gender match*
	Being in hospital can be quite difficult at times.
	The surgeon who the old man on the emergency ward saw silently wished that he could go a little bit faster.
(7d)	*Non c-commanding DP, gender mismatch*
	Being in hospital can be quite difficult at times.
	The surgeon who the old woman on the emergency ward saw silently wished that he could go a little bit faster.

As for QPs in Experiment 2, the DP c-commands the pronoun in (7a,b) but does not in (7c,d). In conditions (7a,c) the pronoun matches in gender with the DP, while in (7b,d) there is a gender mismatch. While variable binding between the pronoun and QP was syntactically illicit in conditions (6c,d) in Experiment 2, there is no constraint that restricts linking the pronoun to the DP in (7c,d) via coreference assignment. As such, if the results of Experiment 2 reflect application of the c-command constraint on variable binding, we expect to find different results with coreference antecedents in Experiment 3. That is, in contrast to the interactions observed in Experiment 2, in Experiment 3 main effects of gender should be observed such that reading times should be longer in gender mismatch conditions (7b,d) than gender match conditions (7a,c), irrespective of c-command.

However, if antecedents inside relative clauses are simply ignored during retrieval as they are not discourse prominent, we expect to observe similar results in Experiment 3 as were observed in Experiment 2. That is, we should observe reliable c-command by gender interactions, with gender mismatch effects being observed in c-command conditions (7a,b) but not non c-commanding conditions (7c,d).

### Methods

#### Participants

32 native English speakers (17 males, mean age 21; range 18–30) from the University of Edinburgh community, none of whom took part in any of the other eye-tracking experiments reported here, took part in Experiment 3. All had normal or corrected-to-normal vision.

#### Materials

The 24 sets of experimental items from Experiment 2 were adapted as in (7). Experimental items were again interspersed with 60 fillers and pseudo-randomly distributed across four presentation lists in a Latin-square design.

#### Procedures

The procedure and data analysis were the same as outlined for Experiment 2.

### Results

Average comprehension question accuracy was 90% (all subjects over 77%). There was no track loss and skipping rates for the pronoun, spillover, prefinal, and final regions were 32, 9, 16, and 12% respectively. Summaries of the reading time data and statistical analysis are provided in Tables 3, 4.

**Table 3:** **Reading times in milliseconds for four eye-movement measures at four regions of texts in Experiment 3 (SDs in parentheses)**.

	**First pass reading time**	**Regression path time**	**Second pass time**	**Total viewing time**
**PRONOUN REGION**				
C-commanding DP, gender match	225 (94)	284 (233)	103 (165)	317 (194)
C-commanding DP, gender mismatch	261 (145)	311 (236)	178 (251)	413 (308)
Non c-commanding DP, gender match	246 (142)	323 (278)	104 (199)	339 (248)
Non c-commanding DP, gender mismatch	261 (144)	316 (262)	147 (247)	410 (305)
**SPILLOVER REGION**				
C-commanding DP, gender match	284 (150)	366 (322)	169 (217)	451 (273)
C-commanding DP, gender mismatch	295 (162)	378 (276)	211 (264)	493 (298)
Non c-commanding DP, gender match	273 (154)	358 (292)	171 (259)	431 (276)
Non c-commanding DP, gender mismatch	283 (175)	383 (338)	209 (331)	510 (377)
**PREFINAL REGION**				
C-commanding DP, gender match	243 (113)	455 (548)	119 (186)	358 (215)
C-commanding DP, gender mismatch	264 (133)	638 (963)	153 (203)	409 (248)
Non c-commanding DP, gender match	259 (143)	420 (536)	129 (219)	384 (263)
Non c-commanding DP, gender mismatch	234 (106)	518 (746)	143 (231)	373 (260)
**FINAL REGION**				
C-commanding DP, gender match	300 (220)	1364 (1391)	83 (212)	400 (299)
C-commanding DP, gender mismatch	293 (177)	1748 (1824)	114 (233)	431 (317)
Non c-commanding DP, gender match	311 (207)	1722 (2160)	92 (224)	426 (352)
Non c-commanding DP, gender mismatch	308 (198)	1783 (2082)	95 (241)	423 (314)

**Table 4:** **Summary of statistical analyses for four eye-movement measures at four regions of texts in Experiment 3**.

	**First pass reading time**		**Regression path time**		**Second pass time**		**Total viewing time**	
	**Estimate**	***t***	**Estimate**	***t***	**Estimate**	***t***	**Estimate**	***t***
**PRONOUN REGION**								
C-command	9 (9)	0.922	15 (31)	0.474	14 (15)	0.952	0 (21)	0.021
Gender	17 (14)	1.261	5 (27)	0.171	58 (19)	3.116^*^	72 (24)	2.999^*^
C-command^*^ gender	20 (21)	0.953	46 (50)	0.907	31 (27)	1.159	34 (40)	0.853
**SPILLOVER REGION**								
C-command	10 (12)	0.783	4 (21)	0.192	1 (20)	0.042	5 (19)	0.234
Gender	11 (12)	0.942	18 (22)	0.842	39 (21)	1.852^(^^*^^)^	59 (21)	2.796^*^
C-command^*^ gender	0 (21)	0.011	8 (48)	0.165	5 (39)	0.141	40 (42)	0.958
**PREFINAL REGION**								
C-command	6 (9)	0.668	78 (51)	1.540	1 (18)	0.046	6 (20)	0.295
Gender	2 (9)	0.209	151 (63)	2.396^*^	22 (16)	1.431	20 (17)	1.174
C-command^*^ gender	43 (18)	2.377^*^	64 (126)	0.512	22 (29)	0.754	59 (37)	1.604
**FINAL REGION**								
C-command	15 (18)	0.830	169 (131)	1.288	5 (15)	0.313	12 (29)	0.423
Gender	7 (18)	0.380	222 (127)	1.749^(^^*^^)^	18 (15)	1.204	13 (21)	0.599
C-command^*^ gender	4 (37)	0.121	266 (257)	1.034	28 (29)	0.975	22 (43)	0.515

Estimate = Model Estimate (SE in brackets). (

* ) = p < 0.10,

* = p < 0.05,

** = p < 0.001.

At the pronoun region, there were significant main effects of gender in second pass and total viewing times, with reading times being longer in gender mismatch conditions (7b,d) compared to gender match conditions (7a,c). In contrast to Experiment 2, there was no hint of an interaction between c-command and gender in any measure at the pronoun region. This suggests that the DP was retrieved irrespective of whether or not it was inside a relative clause. This pattern of results for the second pass times at the pronoun region is shown in Figure 1.

The results of the spillover region replicated this pattern of results. In second pass times there was a marginal main effect of gender, with reading times again tending to be longer following a gender mismatch between the pronoun and DP compared to when there was a gender match. Total viewing times displayed the same pattern of results, with the main effect of gender being fully significant in this measure.

At the prefinal region, there was a significant c-command by gender interaction in first-pass reading times. Here, in the c-command conditions reading times were numerically longer in gender mismatch condition (7b) than gender match condition (7a). The planned comparison was however not significant (*t* = 1.506, *p* = 0.133). The opposite numerical pattern was observed in the two non c-command conditions, with gender match condition (7c) having numerically longer reading times than gender mismatch condition (7d). The planned comparison was however only marginally significant (estimate = 24, SD = 13, *t* = 1.851, *p* = 0.065). It is unclear what this numerical pattern might mean, and it is not replicated in any other measure. Indeed, in the regression path times at this region there was a significant main effect of gender, with reading times following the pattern observed at the pronoun and spillover regions, with reading times being longer following gender mismatches between the DP and pronoun compared to when there was a gender match.

There was also a marginally significant main effect of gender in the regression path times at the final region, with reading times again tending to be longer when the pronoun mismatched in gender with the DP compared to when there was a gender match.

### Discussion

The results of Experiment 3 are in clear contrast to those from Experiment 2. Whereas we observed significant c-command by gender interactions at the pronoun and spillover regions in Experiment 2, in Experiment 3 we observed only significant main effects of gender at these regions. This suggests that, in contrast to Experiment 2, in Experiment 3 participants were equally likely to retrieve the DP antecedent in both the c-command and non c-command conditions. Indeed, the relative time-course of mismatch effects across both experiments is very similar (compare graphs from Experiments 2 and 3 in Figure 1). The crucial difference between the two is that while mismatch effects were restricted to the c-command conditions in Experiment 2, they appear irrespective of c-command in Experiment 3. This provides good evidence that the results of Experiment 2 cannot be explained in terms of antecedents inside relative clauses simply being non-discourse prominent. Rather, while both antecedents that c-command a pronoun and those that do not are readily retrieved, quantified antecedents are only retrieved when variable binding is syntactically licit. It is this contrast between syntactically licit and syntactically illicit pronoun-antecedent dependencies that appears to best explain the contrast in results between Experiments 2 and 3.

Although the results of Experiments 2 and 3 indicate that the c-command constraint restricts antecedent retrieval during language processing, one issue that remains is how the c-command constraint and gender congruence combine during anaphora resolution. In Experiments 2 and 3, there was always at least one gender-matching and syntactically licit antecedent in the discourse, namely the matrix subject DP [*the surgeon* in (5) and (6)]. To fully test how the c-command constraint and gender congruence interact to guide antecedent retrieval, it is also necessary to investigate anaphora resolution when the only syntactically licit antecedent available in the discourse provides only a partial match to the cues at retrieval. Experiment 4 was thus conducted to test this issue.

## Experiment 4

The aim of Experiment 4 was to investigate how the c-command constraint and gender congruence combine to guide antecedent retrieval. Materials in Experiment 4 contained the two non c-command conditions from Experiment 2, additionally manipulating the stereotypical gender relationship between pronoun and matrix subject DP as in (8).

**Table:** 

(8a)	*DP gender match, QP gender match*
	Being in hospital can be quite difficult at times.
	The surgeon who every old man on the emergency ward saw silently wished that he could go a little bit faster.
(8b)	*DP gender match, QP gender mismatch*
	Being in hospital can be quite difficult at times.
	The surgeon who every old woman on the emergency ward saw silently wished that he could go a little bit faster.
(8c)	*DP gender mismatch, QP gender match*
	Being in hospital can be quite difficult at times.
	The surgeon who every old woman on the emergency ward saw silently wished that she could go a little bit faster.
(8d)	*DP gender mismatch, QP gender mismatch*
	Being in hospital can be quite difficult at times.
	The surgeon who every old man on the emergency ward saw silently wished that she could go a little bit faster.

In (8), the QP always appears inside a relative clause and as such is not a syntactically licit antecedent of the pronoun. In each condition, the only syntactically licit antecedent is the matrix subject DP *the surgeon*. In (8a,b), this DP matches in stereotypical gender with the pronoun, whereas in (8c,d) there is a stereotypical gender mismatch. In (8a,c) the non c-commanding QP additionally matches the gender of the pronoun, while in (8b,d) it does not.

Different predictions with regards to the time-course of antecedent retrieval can be made depending on how the c-command constraint and gender congruence combine. If syntactic constraints on anaphora resolution constitute “hard constraints” that gate retrieval to syntactically licit antecedents (Dillon et al., 2013; Chow et al., 2014; Kush et al., 2015), we should observe main effects of the gender of the DP only. Reading times should be longer in DP gender mismatch conditions (8c,d) than in DP gender match conditions (8a,b). The gender of the syntactically illicit QP should not influence reading times at any point in the sentence.

Alternatively, if the c-command constraint and gender congruence combine to guide retrieval, we expect to observe facilitatory interference (e.g., Wagers et al., 2009). In this case, we would expect reading times to generally be longer in DP gender mismatch conditions (8c,d) than DP gender match conditions (8a,b). However, the size of the gender mismatch effect should be reliably attenuated when the structurally illicit QP matches in gender with the pronoun. In this case, reading times should be shorter in condition (8c), when the QP matches the gender of the pronoun, in comparison to (8d), when neither antecedent matches. This result would indicate that when no syntactically licit antecedent is available in the discourse that matches the pronoun's gender, a gender matching but syntactically illicit antecedent may sometimes be retrieved.

Another possibility is that we may observe a difference in the time-course of effects for syntactically licit and illicit antecedents. Sturt (2003) proposed the “defeasible filter” hypothesis which predicts that initially only structurally licit antecedents are considered, but that structurally illicit antecedents can subsequently be retrieved during later stages of processing. Applying this logic to the current experiment, we may observe an initial attempt to retrieve only the syntactically licit DP, followed by subsequent effects of the syntactically illicit QP. In this case, we should observe main effects of stereotypical gender mismatch between the pronoun and DP antecedent only at or shortly after the pronoun, with any effects of the gender of the structurally illicit QP antecedent being in comparison delayed.

### Methods

#### Participants

32 native English speakers (12 males, mean age = 24; range = 18–49) from the University of Edinburgh community with normal or corrected-to-normal vision, none of which took part in Experiments 1–3, took part in Experiment 4.

#### Materials

The 24 experimental items from Experiment 2 were adapted as in (8), and again pseudo-randomly interspersed with 60 fillers across four presentation lists in a Latin-square design. The stereotypical gender manipulations included items that had previously been pre-tested to ensure they displayed the intended stereotypes (Cunnings and Felser, 2013; Cunnings et al., 2014).

#### Procedures

The procedure and data analysis were the same as in Experiments 2 and 3.

### Results

Overall accuracy to comprehension questions was 89% (all subjects above 77%). Track loss accounted for 0.1% of the data. Skipping rates for the pronoun, spillover, prefinal and final regions were 21, 7, 14, and 7% respectively. Summaries of the reading times and statistical analyses are shown in Tables 5, 6.

**Table 5:** **Reading times in milliseconds for four eye-movement measures at four regions of texts in Experiment 4 (SDs in parentheses)**.

	**First pass reading time**	**Regression path time**	**Second pass time**	**Total viewing time**
**PRONOUN REGION**				
DP gender match, QP gender match	251 (137)	356 (413)	96 (173)	332 (230)
DP gender match, QP gender mismatch	253 (124)	291 (185)	99 (184)	361 (278)
DP gender mismatch, QP gender match	256 (121)	334 (321)	149 (226)	405 (258)
DP gender mismatch, QP gender mismatch	253 (145)	312 (286)	144 (231)	375 (271)
**SPILLOVER REGION**				
DP gender match, QP gender match	278 (141)	368 (487)	168 (239)	445 (276)
DP gender match, QP gender mismatch	307 (168)	403 (442)	196 (265)	497 (308)
DP gender mismatch, QP gender match	323 (193)	411 (282)	230 (294)	542 (321)
DP gender mismatch, QP gender mismatch	339 (185)	482 (416)	226 (356)	564 (369)
**PREFINAL REGION**				
DP gender match, QP gender match	266 (144)	459 (507)	146 (182)	396 (228)
DP gender match, QP gender mismatch	279 (133)	556 (862)	144 (214)	410 (257)
DP gender mismatch, QP gender match	272 (128)	522 (682)	182 (255)	441 (271)
DP gender mismatch, QP gender mismatch	286 (163)	587 (845)	157 (228)	434 (269)
**FINAL REGION**				
DP gender match, QP gender match	342 (239)	1717 (1963)	74 (156)	423 (276)
DP gender match, QP gender mismatch	310 (228)	1799 (2285)	96 (220)	437 (327)
DP gender mismatch, QP gender match	338 (235)	2276 (2515)	116 (216)	486 (322)
DP gender mismatch, QP gender mismatch	366 (264)	1736 (1920)	80 (186)	464 (325)

**Table 6:** **Summary of statistical analyses for four eye-movement measures at four regions of texts in Experiment 4**.

	**First pass reading time**		**Regression path time**		**Second pass time**		**Total viewing time**	
	**Estimate**	***t***	**Estimate**	***t***	**Estimate**	***t***	**Estimate**	***t***
**PRONOUN REGION**								
DP gender	4 (10)	0.377	4 (28)	0.144	47 (14)	3.448^**^	46 (19)	2.387^*^
QP gender	1 (12)	0.117	37 (35)	1.069	0 (16)	0.030	3 (20)	0.134
DP gender^*^ QP gender	10 (21)	0.490	32 (57)	0.567	6 (27)	0.231	55 (36)	1.528
**SPILLOVER REGION**								
DP gender	37 (11)	3.209^*^	58 (46)	1.293	45 (21)	2.131^*^	81 (20)	3.968^**^
QP gender	22 (14)	1.617	51 (43)	1.194	12 (20)	0.603	38 (20)	1.884^(^^*^^)^
DP gender^*^ QP gender	12 (27)	0.439	31 (61)	0.503	31 (39)	0.778	36 (39)	0.924
**PREFINAL REGION**								
DP gender	6 (12)	0.480	42 (78)	0.541	24 (15)	1.622	36 (18)	2.003^*^
QP gender	15 (12)	1.262	84 (65)	1.290	13 (20)	0.682	3 (19)	0.146
DP gender^*^ QP gender	3 (25)	0.134	25 (122)	0.208	22 (33)	0.645	14 (35)	0.410
**FINAL REGION**								
DP gender	28 (17)	1.636	247 (125)	1.979^*^	10 (14)	0.709	48 (21)	2.261^*^
QP gender	7 (22)	0.303	250 (135)	1.846^(^^*^^)^	5 (13)	0.391	11 (21)	0.539
DP gender^*^ QP gender	61 (33)	1.864^(^^*^^)^	627 (297)	2.109^*^	58 (37)	1.579	32 (49)	0.657

Estimate = Model Estimate (SE in brackets). (

* ) = p < 0.10,

* = p < 0.05,

** = p < 0.001.

At the pronoun region, we observed significant main effects of the gender of the DP in both second pass and total viewing times. In both measures, reading times were longer when the DP mismatched in stereotypical gender with the pronoun, as in (8c,d) compared to when there was a stereotypical gender match, as in (8a,b). The gender of the QP did not significantly affect reading times in any measure at this region. These results suggest that participants attempted to retrieve the syntactically licit DP antecedent. This pattern of results is illustrated in Figure 1, which shows the second pass times at the pronoun region.

At the spillover region, there was a significant main effect of DP gender in first pass times. Here, reading times were again longer when the DP mismatched in stereotypical gender with the pronoun compared to when there was a gender match. There was also a significant main effect of the gender of the DP in both second-pass and total viewing times, with reading times again being longer when the DP mismatched in stereotypical gender with the pronoun. There was additionally a marginally significant main effect of QP gender in total viewing times only. Here, reading times tended to also be longer when the QP mismatched in gender with the pronoun. There was no hint of an interaction however, as this numerical trend for longer reading times following gender mismatching QPs was observed in both the DP match and DP mismatch conditions.

At the prefinal region, there was again a significant main effect of DP gender in total viewing times, with reading times being longer when the DP mismatched in stereotypical gender with the pronoun. No significant effects of the gender of the QP were found at this region.

At the final region there was a marginally significant interaction in first-pass times. Here, in the DP stereotypical gender match conditions, reading times tended to be longer in QP match condition (8a) compared to QP mismatch condition (8b), but the planned comparison was not significant (*t* = 1.476, *p* = 0.141). The opposite numerical pattern was observed in the DP stereotypical gender mismatch conditions, but again the comparison was not significant (*t* = 0.779, *p* = 0.437). In regression path times the main effect of the stereotypical gender of the DP was significant, the main effect of the gender of the QP marginal, and the DP gender by QP gender interaction significant. In this measure, while reading times in DP match conditions (8a,b) did not differ (*t* = 0.381, *p* = 0.703), for the DP stereotypical gender mismatch conditions, reading times were longer in QP match condition (8c) than QP mismatch condition (8d) (estimate = 569, SD = 220, *t* = 2.591, *p* = 0.010). While this reading time measure thus provides evidence of the QP's gender significantly influencing reading times, the direction of the effect in the DP stereotypical gender mismatch conditions is in the *opposite* direction to that predicted by facilitatory interference. Total viewing times at the final region exhibited reading times similar to earlier regions of text, with reading times being significantly longer when the DP mismatched in stereotypical gender with the pronoun compared to when there was a gender match. The QP did not significantly influence reading times in this measure.

### Discussion

The results of Experiment 4 indicate that readers readily retrieved the syntactically licit DP antecedent upon encountering the pronoun. In a number of measures across all regions of text reported, we observed significantly longer reading times when the DP mismatched in stereotypical gender with the pronoun compared to when there was a gender match. Effects of the gender of the QP antecedent were more elusive and the one significant effect that we did observe was delayed in comparison to the effects that were observed of the DP's gender. While DP stereotypical gender mismatch effects were first observed in second pass and total viewing times at the pronoun, and first pass times at the spillover region, the only reliable effect of the gender of the QP was observed in the regression path times at the final region. We leave discussion of this delayed effect of the QP's gender until the General Discussion, but overall interpret the relative time-course of effects as indicating that the c-command constraint on variable binding restricts the initial stages of antecedent retrieval during comparatively earlier stages of anaphora resolution. We discuss the implications of these results, along with the other experiments reported above, in more detail below.

## General discussion

The aim of this study was to investigate if the c-command restriction on variable binding restricts antecedent retrieval during anaphora resolution. The results of Experiment 1 indicate that native English speakers are sensitive to the c-command restriction on binding by quantified antecedents in an offline judgment task. Experiment 2, which investigated the extent to which QP antecedents are retrieved upon encountering a pronoun during online processing, indicates that participants readily retrieved the QP upon encountering the pronoun, but only when the QP c-commanded the pronoun. The results of Experiment 3 showed that retrieval of DP antecedents, which is not contingent on c-command, was equally likely irrespective of whether or not the DP c-commanded the pronoun. The results of Experiments 2 and 3 together confirm that it is not the case that non c-commanding antecedents are generally ignored due to their lower discourse salience. Instead, both c-commanding and non c-commanding antecedents are readily retrieved, but only when they are syntactically licit antecedents for a pronoun. Finally, the results of Experiment 4 indicate that when only one syntactically licit antecedent is available in the discourse, that antecedent is preferentially retrieved over a syntactically illicit QP, even when the syntactically licit antecedent mismatches in gender with the pronoun. This different pattern of results across the three eye-movement experiments is illustrated in Figure 1. Together, these data indicate that the c-command constraint on variable binding restricts antecedent retrieval during anaphora resolution. Below we discuss the implications of these results with regards to how the c-command constraint on variable binding may be implemented in models of memory retrieval, and the relative weightings of different cues to antecedent retrieval during anaphora resolution.

### Implementing the C-command constraint

One potential way to help ensure that only syntactically licit QPs are retrieved during anaphora resolution might be to restrict at least initial memory access operations to antecedents that c-command a pronoun. This proposal would be similar to claims in the linguistics literature that variable binding is computed before coreference assignment (e.g., Reuland, 2001, 2011; Koornneef, 2008). In the current study, c-commanding antecedents always appeared in the main clause of the critical sentence, while non c-commanding antecedents appeared in relative clauses. The preference for retrieving a c-commanding antecedent in the current study could thus potentially be achieved by postulating that pronouns preferentially cue retrieval of an antecedent carrying a [+main clause] feature. However, this would also predict that non c-commanding DPs, even though they can be linked to the pronoun via coreference assignment, should also initially be ignored. Note however that while we observed selective retrieval of QPs in Experiment 2 and retrieval of DPs irrespective of c-command in Experiment 3, the time-course of gender mismatch effects across the pronoun and spillover regions in both experiments was very similar. If an initial retrieval favors antecedents carrying the [+main clause] feature only, we would have expected to see a delay in gender mismatch effects for non c-commanding DP antecedents compared to c-commanding DP antecedents in Experiment 3 that was not observed. The results from Cunnings et al. (2014) also clearly indicate that there is no initial preference for c-commanding over non c-commanding antecedents. Thus, we believe the hypothesis that initial retrieval operations should always simply ignore non c-commanding antecedents can be rejected. Nor can the retrieval operation initially target *only* referential antecedents or quantified ones, considering that Cunnings et al. (2014) observed no overall preference for either variable binding or coreference assignment.

Sensitivity to the c-command constraint on variable binding thus requires a restriction that selectively retrieves antecedents based on the c-command relationship between the pronoun and QP. As noted in the introduction, some have claimed that this type of *relational* constraint may be difficult to implement in content-addressable memory architectures (Kush, 2013; Kush et al., 2015). Kush et al. propose that one way to implement the c-command constraint on variable binding would be to encode all potential antecedents with an ACCESSIBLE feature that the parser is able to dynamically update based on the current state of the parse during incremental processing. That is, antecedents are always initially marked as [+accessible], but retrieval operations at specific points during an incremental parse may deactivate this feature if need be. We believe this proposal could account for our results as follows. In Experiments 2–4, the critical QP/DP (*every old man/woman; the old man/woman*) will initially be encoded as being [+accessible]. In the c-commanding QP/DP conditions, this feature will always remain activated. In the non c-commanding QP/DP conditions, upon reaching the right-most edge of the relative clause, a retrieval operation will access all antecedents within the relative clause, deactivating the ACCESSIBLE feature for QPs to ensure that they are no longer possible targets for retrieval, but leaving it unchanged for DPs. In this way, well-known clause “wrap-up” effects may in part involve updating items in a particular clause as being either accessible or inaccessible to further retrieval operations. Upon encountering the pronoun, the ACCESSIBLE feature will be a highly weighted cue to retrieval, activating DPs irrespective of c-command, but activating c-commanding QPs only.

### Cue weighting during anaphora resolution

The results of the current study indicate that the c-command constraint on variable binding, perhaps implemented using the ACCESSIBLE feature as above, is a highly weighted cue to antecedent retrieval. The gender mismatch effects observed in Experiment 2 indicate that participants will readily retrieve a c-commanding QP during processing, but we found no evidence of the QP being retrieved when it did not c-command the pronoun. In Experiment 4, when the QP was always syntactically illicit, we found that a number of reading time measures were significantly affected by the stereotypical gender of the syntactically licit DP only. The earliest measures where we observed this effect were those including first pass processing at the spillover region and second pass processing at the pronoun region.

Some models of memory retrieval assume that cues combine in an equally-weighted fashion to guide retrieval during language processing (e.g., Lewis et al., 2006). Evidence from facilitatory interference effects during subject-verb agreement processing for example, suggest that for at least some dependencies syntactic constraints and agreement markers are equally weighted cues to retrieval (e.g., Wagers et al., 2009). More recently however it has been claimed that retrieval cues during language processing are not always equally weighted (Van Dyke and McElree, 2011; Dillon et al., 2013). For example, Dillon et al. claimed that syntactic binding constraints constitute “hard constraints” that restrict retrieval to syntactically licit antecedents. We argued that the most obvious kind of evidence that the c-command constraint and gender congruence combine equally to guide retrieval would be from facilitatory interference effects similar to those observed for subject-verb agreement. However, we failed to observe this pattern of results in Experiment 4.

Although we failed to observe facilitatory interference, Badecker and Straub (2002) reported a different type of *inhibitory* interference in a series of self-paced reading experiments. They observed longer reading times when multiple antecedents matched in gender with a reflexive or pronoun compared to when there was only one gender matching antecedent. Such effects could indicate that when there are multiple gender matching antecedents in the discourse, both syntactically licit and illicit antecedents compete for retrieval. The clearest evidence of this type of interference in the current study would have been from longer reading times in Experiment 4 in multiple gender match condition (8a) compared to the single match condition (8b). However, we also failed to observe this type of effect.

The clearest evidence of the gender of the QP significantly affecting reading times that we did observe was in the *opposite* direction predicted by facilitatory interference, and was also dissimilar to the effects observed by Badecker and Straub (2002). In the regression path times for the final region in Experiment 4, reading times were significantly longer when the syntactically illicit QP matched the gender of the pronoun, but only when the grammatically licit DP antecedent itself mismatched in gender with the pronoun. Note also that this effect of the QP's gender appears delayed in comparison to the significant main effects of the stereotypical gender of the syntactically licit DP.

In line with recent proposals that not all cues to memory retrieval are equally weighted during language processing (Van Dyke and McElree, 2011; Dillon et al., 2013), we argue that the c-command constraint on variable binding, implemented with the ACCESSIBLE feature, is a more highly weighted cue to antecedent retrieval than gender congruence during anaphora resolution. Whether or not the c-command restriction acts as a “hard constraint” that imposes a categorical ban on the retrieval of syntactically illicit antecedents is difficult to conclude. However, the relative time-course of effects observed for DP and QP antecedents in Experiment 4 may bear on this issue. Recall that in the DP stereotypical gender mismatch conditions in the regression path times of the final region in Experiment 4, we observed *longer* reading times when the QP matched the gender of the pronoun compared to when it mismatched. We remain cautious in interpreting precisely what this effect may index, but it could potentially indicate that readers sometimes attempted to coerce an interpretation in which the pronoun was linked to a syntactically illicit but gender matching QP antecedent, with this coercion of a syntactically illicit interpretation leading to longer reading times. The time-course of this effect, appearing at the sentence final region and delayed in comparison to stereotypical gender violations between the DP and pronoun, may indicate that it reflects a relatively late interpretive process that tries to coerce an otherwise dispreferred interpretation for the pronoun. Similar to Sturt's (2003) defeasible filter hypothesis, we propose that the time-course of effects observed in Experiment 4 may indicate that initially, retrieval operations attempt to retrieve syntactically licit antecedents only. Readers may sometimes try to coerce syntactically illicit interpretations during comparatively later stages of processing however, perhaps during reanalysis after initially retrieving a syntactically licit, but gender-mismatching antecedent. We note also however that other interpretations of this delayed effect are possible. Kush et al. (2015) for example, found that non c-commanding QPs did not influence reading times during early stages of anaphor resolution in sentences like (4b), but did find some suggestive evidence of delayed effects of non c-commanding QPs influencing processing in measures that included second-pass processing. They claimed that such delayed effects might index coercion of an additional referential antecedent for the pronoun from the set of antecedents implied by the quantifier. In this sense, when the pronoun matches the gender of a non c-commanding QP in sentences like (8c), the delayed effect we observed may index coercion of a referential antecedent (*an old woman*) from the set of antecedents implied by the QP (*every old woman*). We do not attempt to tease apart these two interpretations here. Irrespective of how these effects are to be interpreted, as they appear delayed in comparison to effects of syntactically licit antecedents, we maintain that retrieval operations initially attempt to retrieve grammatically licit antecedents only.

Finally, we note that counterexamples in which variable binding appears to be possible between a pronoun and antecedent irrespective of c-command have been discussed in the linguistics literature. For example, in *Every boy's mother says that he is special* the pronoun can be bound by the QP *every boy* even though the QP does not c-command the pronoun under the standard definition. Barker (2012) discusses a number of such counterexamples and claims that the restriction on variable binding should be recast in terms of semantic scope rather than c-command. The relative clause manipulation tested in the current study is a relatively clear-cut case where both traditional accounts and Barker would predict that variable binding is not permitted. Our results show that variable binding is not attempted during processing in such cases, at least during early stages of antecedent retrieval (see also Kush et al., 2015). The extent to which pronouns may trigger retrieval of non c-commanding QPs in other constructions is less well understood. Some researchers have investigated whether pronouns are linked to QPs in non c-commanding configurations other than the relative clause manipulation in the current study (e.g., Carminati et al., 2002; Kush et al., 2015, Experiment 1c), but these experiments used different diagnostics for dependency formation and did not use interference paradigms as in Experiment 4 here. One question that arises is whether retrieval of QPs upon encountering a pronoun is always, at least initially, restricted to c-commanding quantified antecedents, or whether in exceptional cases, as in sentences like *Every boy's mother says that he is special*, quantified antecedents are always accessible. How the c-command constraint and gender/number congruence interact to guide anaphora resolution in other constructions will thus be an important avenue of further research to investigate the extent to which the current findings generalize beyond the relative clauses tested here.

## Conclusion

Across four experiments we investigated how constraints on pronoun interpretation influence the retrieval of quantified and non-quantified antecedents in different syntactic configurations. We found that variable binding between a pronoun and quantified antecedent was only attempted if the quantifier c-commanded the pronoun. Retrieval of coreference antecedents, which is not contingent on c-command, was attempted irrespective of c-command. We interpret these results as indicating that syntactic constraints restrict memory retrieval operations during anaphora resolution. We conclude that the c-command constraint on variable binding constitutes a highly weighted cue during anaphora resolution that, at least initially, guides retrieval operations to access syntactically licit antecedents only.

### Conflict of interest statement

The authors declare that the research was conducted in the absence of any commercial or financial relationships that could be construed as a potential conflict of interest.

## Footnotes

^1^The participants in Experiment 1 also completed Experiment 3. All participants completed Experiment 3 before Experiment 1.

^2^A reviewer notes that the results of Experiment 1 on their own could equally be explained in terms of a dispreference for linking pronouns to antecedents inside relative clauses, irrespective of quantification, rather than a specific constraint on variable binding to QPs. While this is a possible explanation of the results in Experiment 1, the results of Experiments 2 and 3 suggest that the restriction on binding to QPs is best characterised in terms of the c-command constraint, rather than a general dispreference against antecedents inside relative clauses.

^3^Skipping rates at the pronoun region were quite high in Experiments 2–4. We thus conducted an additional analysis in which the two first-pass measures at the pronoun were calculated using a leftward-shifting procedure (see Sturt, 2003, p. 548). In this analysis, if the pronoun was initially skipped during reading, fixations up to four characters to the left of the region boundary were included in the calculation of first-pass and regression path times. This reduced skipping rates at the pronoun to below 8% across experiments, but did not alter the overall pattern of results compared to the non-shifted analysis reported in the main text.

## Appendix A

The materials for Experiment 1 are provided below. The c-command manipulation is denoted in square brackets, delimited with a forward slash (/).

1. The soldier [suspected that every old man at the bar/who every old man at the bar suspected] suddenly wished that he had drunk a bit less that afternoon.
2. The footballer [observed that every salesman in the shop/who every salesman in the shop observed] secretly thought that he could offer some advice.
3. The butcher [heard that every boy in the street/who every boy in the street heard] loudly said that he could not lift the heavy box.
4. The farmer [saw that every schoolboy in the field/who every schoolboy in the field saw] truly feared that he would disturb the wildlife.
5. The pilot [knew that every waiter in the café/who every waiter in the café knew] really hoped that he would avoid getting ill.
6. The sailor [trusted that every prince at the royal celebration/who every prince at the royal celebration trusted] clearly understood that he should not join in the dance.
7. The surgeon [suggested that every man on the waiting list/who every man on the waiting list suggested] definitely realized that he needed some help.
8. The builder [noticed that every father at the school gate/who every father at the school gate noticed] openly stated that he should get back to work soon.
9. The nurse [noticed that every old woman in the hospital/who every old woman in the hospital noticed] genuinely expected that she would be allowed to leave very soon.
10. The secretary [saw that every saleswoman in the meeting/who every saleswoman in the meeting saw] obviously worried that she was not making a contribution.
11. The babysitter [observed that every girl in the house/who every girl in the house observed] thoroughly wished that she could reach the biscuit tin.
12. The kindergarten teacher [trusted that every schoolgirl in the class/who every schoolgirl in the class trusted] earnestly believed that she could improve during the term.
13. The housekeeper [knew that every waitress at the event/who every waitress at the event knew] rightly realized that she could create a good atmosphere.
14. The cleaner [suspected that every princess in the castle/who every princess in the castle suspected] truly thought that she could find the lost set of keys.
15. The typist [heard that every woman in the office/who every woman in the office heard] suddenly decided that she should leave early that day.
16. The florist [suggested that every mother on the committee/who every mother on the committee suggested] sincerely hoped that she could get the flowers in time.

## Appendix B

The materials for Experiment 2 are provided below. Gender manipulations are shown in parenthesis and square brackets denote the c-command manipulation, delimited with a forward slash (/).

1. Being in hospital can be quite difficult at times. The surgeon [saw that every old man (woman) on the emergency ward/who every old man (woman) on the emergency ward saw] silently wished that he could go a little bit faster.
2. A fight had broken out at the celebration dinner! The footballer [noticed that every waiter (waitress) in the hotel/who every waiter (waitress) in the hotel noticed] quickly realized that he should move away from the table.
3. There was only one way to sneak into the palace party. The soldier [suspected that every prince (princess) outside the castle/who every prince (princess) outside the castle suspected] honestly doubted that he could climb over the high wall.
4. The company was spending a lot of money on a new building. The builder [suggested that every businessman (businesswoman) in the firm/who every businessman (businesswoman) in the firm suggested] sincerely thought that he could do the job much better.
5. There had been a big fire at the local high-school. The firefighter [heard that every father (mother) in the crowd/who every father (mother) in the crowd saw] quickly declared that he should be allowed to go back.
6. The tour of the new local airport was very enjoyable. The pilot [saw that every boy (girl) near the large plane/who every boy (girl) near the large plane saw] clearly expected that he would go up the steps first.
7. There was an uneasy atmosphere after the big match. The boxer [trusted that every policeman (policewoman) in the room/who every policeman (policewoman) in the room trusted] soon realized that he could reach the back exit safely.
8. Trading always starts very early during the morning. The butcher [suspected that every salesman (saleswoman) at the market/who every salesman (saleswoman) at the market suspected] secretly hoped that he could decide on the new price.
9. It was an extremely hot afternoon, even out at sea. The sailor [believed that every schoolboy (schoolgirl) on the boat/who every schoolboy (schoolgirl) on the boat believed] genuinely thought that he could jump into the cold water.
10. It's not very good to be selfish all of the time. The farmer [knew that every little boy (girl) in the small village/who every little boy (girl) in the small village knew] slowly realized that he must share the apples from the orchard.
11. Water had been pouring into the kitchen for a whole day. The plumber [suggested that every young man (woman) in the house/who every young man (woman) in the house suggested] seriously thought that he could stop the leak very easily.
12. It is unusual to talk openly about personal finances. The priest [heard that every man (woman) at the village church/who every man (woman) at the village church heard] suddenly claimed that he should give more money to charity.
13. Modern life can be a little bit too busy at times. The cleaner [knew that every old woman (man) in the care-home/who every old woman (man) in the care-home knew] truly wished that she had much more time to talk.
14. There had been a lot of changes to the menu that night. The fashion model [heard that every waitress (waiter) in the restaurant/who every waitress (waiter) in the restaurant heard] incorrectly feared that she would get the wrong evening meal.
15. It was the beginning of another very important day. The housekeeper [trusted that every princess (prince) in the old castle/who every princess (prince) in the old castle trusted] clearly understood that she must make a very good impression.
16. There were going to be some big changes at the large company. The secretary [suspected that every businesswoman (businessman) at the meeting/who every businesswoman (businessman) at the meeting suspected] correctly realized that she could change the terms of the contract.
17. Life can get very tiring if you don't find time to rest. The babysitter [trusted that every mother (father) in the big group/who every mother (father) in the big group trusted] eventually realized that she needed to relax now and again.
18. The flower festival was always a very busy time of year. The florist [believed that every girl (boy) in the beautiful garden/who every girl (boy) in the beautiful garden believed] wishfully thought that she could smell all the lovely roses.
19. The carnival always seems to get bigger every year. The nurse [knew that every policewoman (policeman) at the annual event/who every policewoman (policeman) at the annual event knew] fully understood that she must help with any big emergency.
20. Working hard can sometimes bring great rewards. The typist [noticed that every saleswoman (salesman) in the firm/who every saleswoman (salesman) in the firm noticed] never doubted that she would get a big bonus payment.
21. The baseball match was due to start in 2 h. The cheerleader [noticed that every schoolgirl (schoolboy) in the team/who every schoolgirl (schoolboy) in the team noticed] quietly expected that she would stand in the front row.
22. It was yet another really hectic day at the theater in town. The beautician [saw that every little girl (boy) in the glamorous show/who every little girl (boy) in the glamorous show saw] mistakenly worried that she would mess up the expensive costumes.
23. There had been a lot of rumors going around for a while. The fortune-teller [believed that every young woman (man) at the fair/who every young woman (man) at the fair believed) unexpectedly claimed that she was not telling the whole story.
24. It can sometimes be very difficult to ask for assistance. The kindergarten teacher [suggested that every woman (man) in the village/who every woman (man) in the village suggested] wrongfully thought that she could manage without any extra help.
